# Clinical Characteristics and Long-Term Outcomes of Young Women With Breast Cancer: A Multicenter Study From Uruguay

**DOI:** 10.7759/cureus.103645

**Published:** 2026-02-15

**Authors:** Natalia Camejo, Dahiana Amarillo, Cecilia Castillo, Carla Olguin, Maria Navas, Micaela Mendez, Antonella Duran, Yamila Firpo, Tatiana Gordienko, Guadalupe Herrera, Isabel Alonso, Gabriel Krygier

**Affiliations:** 1 Clinical Oncology, Hospital de Clínicas “Dr. Manuel Quintela”, Montevideo, URY; 2 Clinical Oncology, School of Medicine, University of the Republic, Montevideo, URY; 3 Academic Unit of Preventive and Social Medicine, School of Medicine, University of the Republic, Montevideo, URY; 4 Clinical Oncology, Centro Hospitalario Pereira Rossell, Montevideo, URY

**Keywords:** breast cancer, latin america, real-word data, survival outcomes, young adult women

## Abstract

Background: Breast cancer in women aged ≤40 years is uncommon but clinically challenging, often associated with aggressive pathological features and advanced-stage diagnosis. In Latin America, real-world data on long-term outcomes in this population remain limited. This study aimed to describe clinicopathological characteristics, treatment patterns, and survival outcomes of young women with breast cancer treated in Uruguay.

Methods: We conducted a multicenter retrospective cohort study including women aged 18-40 years diagnosed with invasive breast cancer between 2006 and 2024 at two public referral centers. Clinical, pathological, and treatment data were collected from medical records. Overall survival (OS) and disease-free survival (DFS) were estimated using Kaplan-Meier analysis.

Results: A total of 267 patients were included (mean age at diagnosis, 34.8 years). Invasive ductal carcinoma was the predominant histology (85.8%), with a high proportion of grade III tumors (39.3%). The most common biological subtype was luminal (48.8%), followed by HER2-positive (27.7%) and triple-negative disease (17.2%). Stage II disease was most frequent (40.4%), while 28.7% of patients presented with stage III disease. Modified radical mastectomy was the most commonly performed surgical procedure (38.2%). Most patients received surgery, radiotherapy, and systemic therapy according to recurrence risk. With a median follow-up of 52.3 months, estimated OS was 84.8% at 5 years and 80.9% at 10 years. Survival outcomes differed significantly according to clinical stage but not biological subtype.

Conclusions: Young women with breast cancer in Uruguay frequently present with aggressive tumor features and advanced disease at diagnosis. Nevertheless, when managed within a multidisciplinary framework and treated according to current standards of care, long-term survival outcomes are favorable and comparable to international reports. These findings underscore the importance of early detection strategies and equitable access to guideline-based care in middle-income countries.

## Introduction

Breast cancer diagnosed before the age of 40 is clinically defined as breast cancer in young women, with 40 years widely accepted as the cutoff for this age group [1,2]. This definition is clinically relevant, as young women with breast cancer exhibit distinct clinical, biological, and prognostic characteristics compared with older patients.

Globally, breast cancer in young women accounts for approximately 10%-15% of all cases, although this proportion varies according to geographic region and population age structure [3]. While the absolute incidence is lower than in older women, the relative burden is higher in regions with younger populations, such as Latin America. Population-based studies and systematic reviews have reported that 20%-27% of breast cancer cases in this region are diagnosed in women younger than 45 years, nearly twice the proportion observed in high-income countries [4,5]. National cancer registries from countries such as Brazil, Colombia, Costa Rica, and Ecuador confirm this pattern and describe a progressive increase in both incidence and mortality among young women, representing a growing challenge for health systems across the region [4-6].

Moreover, the literature indicates that both the incidence and mortality of breast cancer in young women are increasing in Latin America, representing a growing challenge for health systems across the region [4-6]. The clinical relevance of distinguishing this age subgroup lies in several aspects. Biologically, breast cancer in young women is associated with more aggressive features, including a higher prevalence of triple-negative and HER2-positive subtypes, higher histologic grade, increased tumor proliferation, and a greater proportion of advanced-stage disease at diagnosis [2,7-9].

From a prognostic standpoint, women younger than 40 years have worse overall survival (OS) and disease-free survival (DFS) compared with older patients, even within the same molecular subtypes, with a particularly increased risk of recurrence in hormone receptor-positive tumors [2,7,9,10]. In addition, this group shows a higher prevalence of germline mutations in cancer predisposition genes, such as BRCA1 and BRCA2, underscoring the importance of genetic testing and risk-reduction strategies for both patients and their families [2,11].

From a diagnostic and therapeutic perspective, the exclusion of young women from population-based screening programs, together with clinical presentations that may be mistaken for physiological changes related to pregnancy or lactation, contributes to later-stage diagnoses. Therapeutic management is often more complex and may involve greater use of chemotherapy, along with specific considerations related to fertility preservation, pregnancy, and the long-term effects of treatment [2,8,9]. Although no molecular subtype exclusive to young age has been identified, available evidence suggests that breast cancer in this group represents a distinct clinical entity with specific research and management needs [1,2,7,10].

Breast cancer in young women therefore constitutes a particular clinical and social challenge, with substantial impact at both the individual and family levels. In addition to presenting with biologically more aggressive tumors and poorer long-term outcomes, these patients face age-specific issues, including fertility preservation, psychosocial distress associated with the diagnosis, and long-term treatment-related consequences. As many are in their reproductive and working years, management should integrate not only oncologic considerations but also reproductive, psychosocial, and socioeconomic aspects, underscoring the need for a multidisciplinary and comprehensive approach in this population [12,13].

The aim of this study was to describe the frequency, clinicopathological characteristics, and survival outcomes of breast cancer in young women treated in Uruguay between 2006 and 2024.

## Materials and methods

Materials and methods

A retrospective observational study was conducted, including women diagnosed with breast cancer before the age of 40 who were treated at the Oncology Services of Hospital de Clínicas and Centro Hospitalario Pereira Rossell between January 1, 2006, and December 31, 2024. Eligible participants were women aged 18-40 years with histologically confirmed invasive breast adenocarcinoma who provided informed consent. Data were collected through a review of medical records, including electronic records and institutional patient files. Survival information was obtained from clinical follow-up documented in medical records; no systematic telephone follow-up was performed. Family screening of relatives was not included in this study.

Statistical analysis

The frequency of breast cancer in women aged ≤40 years was expressed as absolute numbers and relative frequencies (%) of the total cases registered at each institution. Clinicopathological characteristics were summarized using descriptive statistics. Survival outcomes were estimated using the Kaplan-Meier method, and differences between groups were assessed with the log-rank test. DFS was defined as the time from diagnosis to disease recurrence or death, and OS as the time from diagnosis to death from any cause. A Cox proportional hazards regression model was used to evaluate factors associated with mortality.

Statistical analyses were performed using JASP (University of Amsterdam, Amsterdam, Netherlands) and R (R Foundation for Statistical Computing, Vienna, Austria, https://www.R-project.org/). Tables and figures were generated using Google Sheets (Google, Mountain View, CA, USA) and R.

Ethical considerations

The study was approved by the Ethics Committees of Hospital de Clínicas and Centro Hospitalario Pereira Rossell and registered with the Ministry of Public Health. It was conducted in accordance with the Declaration of Helsinki, Decree 158/019, and Law 18.331 on Personal Data Protection. Data confidentiality was maintained through anonymization, and all information was used exclusively for scientific purposes. All participants provided written informed consent prior to inclusion in the study.

## Results

A total of 267 women were included. The mean age at diagnosis was 34.8 years (range, 22-40). A significant family history of breast and/or ovarian cancer was present in 27.7% of patients (n = 71), defined as three or more cases of breast or ovarian cancer with at least one diagnosed before age 50, or two or more cases with at least one diagnosed before age 50 and/or Ashkenazi Jewish ancestry. Germline genetic testing for hereditary cancer predisposition was performed in 4.9% of patients (n = 13); among those tested, 76.9% (n = 10) harbored pathogenic variants in BRCA1 or BRCA2.

Risk-reducing salpingo-oophorectomy was not systematically offered to all patients and was considered individually, primarily in those with confirmed germline BRCA1/2 pathogenic variants, according to clinical guidelines and availability at the time of diagnosis. Data on prophylactic surgeries were not consistently available and were therefore not included as a study variable.

During follow-up (median, 52.3 months), 23.5% of patients (n = 63) developed a second primary malignancy: 19.5% (n = 52) were non-breast cancers, and 4.1% (n = 11) corresponded to a second primary breast cancer. Genomic platforms (Oncotype DX, MammaPrint, or others) were used in 21.0% of patients (n = 56).

Stage II disease was the most frequent at diagnosis (25.8%, n = 69), followed by stage III (18.4%, n = 49). Regarding histologic grade, grade III tumors predominated (39.3%, n = 105), followed by grade II tumors (31.8%, n = 85).

Based on hormone receptor and HER2 expression, the luminal subtype was the most common (48.8%, n = 133), followed by HER2-positive disease (27.7%, n = 74) and triple-negative breast cancer (17.2%, n = 46) (Table 1).

**Table 1:** Clinical and pathological characteristics of the included patients (N = 267)

Characteristic	n (%)
Age at diagnosis (years), median (range)	34.8 (22–40)
Stage 0	1 (0.4)
Stage I	26 (9.7)
Stage II	69 (25.8)
Stage III	49 (18.4)
Stage IV	26 (9.7)
Stage not available	96 (36.0)
Invasive ductal carcinoma	229 (85.8)
Invasive lobular carcinoma	12 (4.5)
Mixed histology	1 (0.4)
Other histological types	5 (1.9)
Histological type not available	12 (4.5)
Histological grade I	12 (4.5)
Histological grade II	85 (31.8)
Histological grade III	105 (39.3)
Histological grade not available	65 (24.3)
Luminal subtype	133 (48.8)
HER2-positive subtype	74 (27.7)
Triple-negative subtype	46 (17.2)
Biological subtype not available	14 (5.2)

Regarding surgical management and axillary approach, modified radical mastectomy was the most frequently performed procedure (38.2%, n = 102), followed by mastectomy with sentinel lymph node biopsy (22.1%, n = 59) and breast-conserving surgery with sentinel lymph node biopsy (16.1%, n = 43). Bilateral mastectomy was not routinely offered at the time of primary surgery. Surgical decisions were individualized based on tumor characteristics, genetic risk assessment when available, patient preference, and institutional practice at the time of diagnosis. Breast reconstruction was performed in 43.4% of patients (n = 116) at the time of surgery.

Overall, 38.5% of patients (n = 103) received adjuvant chemotherapy. The most commonly used regimens were sequential or concurrent anthracycline and taxane combinations (29.7%, n = 41), followed by doxorubicin/cyclophosphamide (29.7%, n = 41) and docetaxel/cyclophosphamide (25.4%, n = 35). The decision to administer adjuvant chemotherapy was based on recurrence risk, tumor biology, and clinical stage, according to national and international guidelines in effect at the time of diagnosis. Variability in chemotherapy use reflects changes in treatment standards over the study period rather than differences in treatment accessibility. All patients with HER2-positive tumors received adjuvant trastuzumab.

Adjuvant radiotherapy was administered to 83.5% of patients (n = 223). In addition, 57.0% of patients (n = 152) received adjuvant endocrine therapy, most commonly tamoxifen for 5 years (48.8%, n = 109), followed by sequential tamoxifen and aromatase inhibitors for 5 years (6.7%, n = 15).

**Figure 1:**
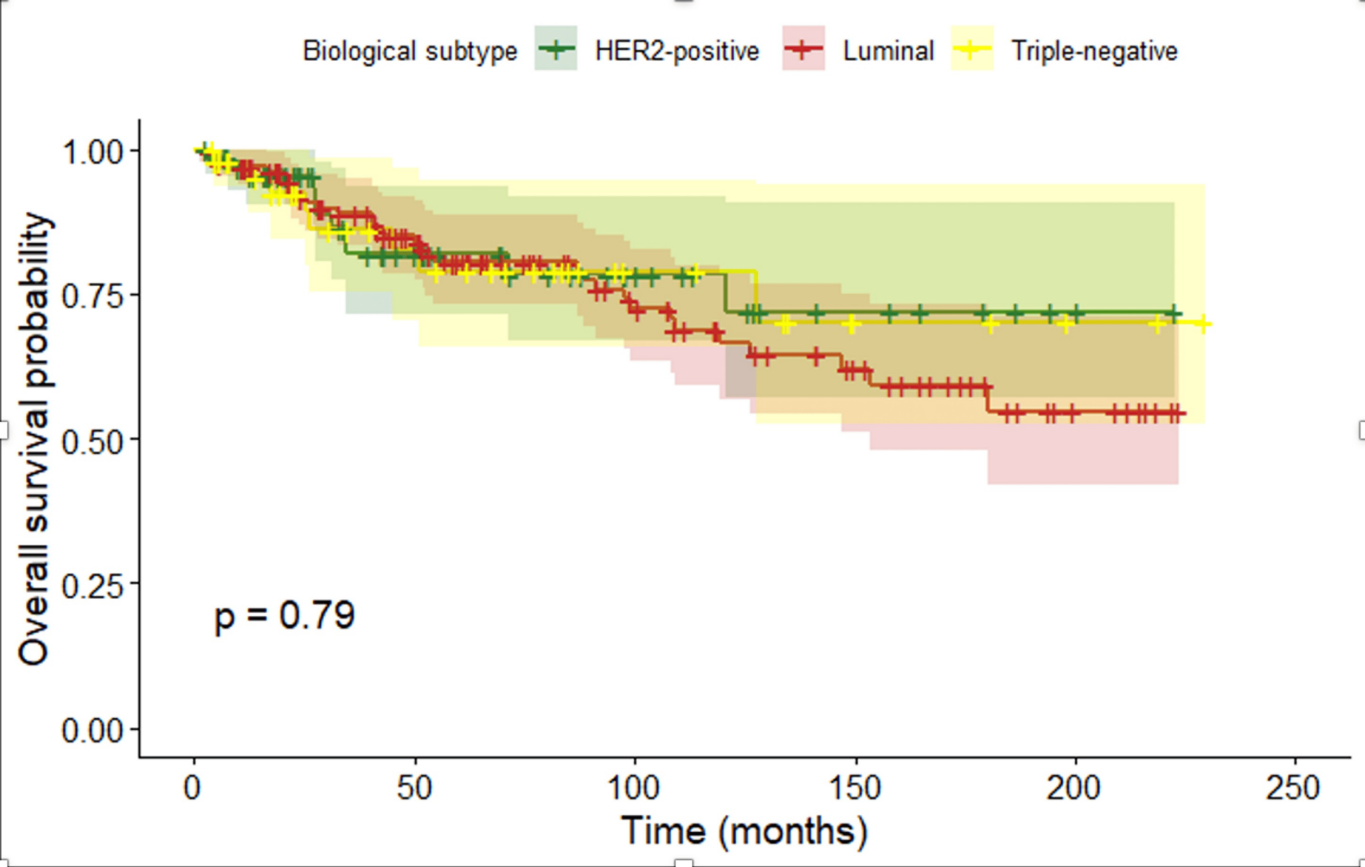
Overall survival according to biological subtype

Regarding OS, with a median follow-up of 52.3 months (95% CI, 43.3-65.2), Kaplan-Meier analysis showed high and sustained survival over time. The estimated OS was 84.8% at 5 years and 80.9% at 10 years. When OS was analyzed according to biological subtype, no statistically significant differences were observed between groups (p = 0.79) (Figure 1).

**Figure 2:**
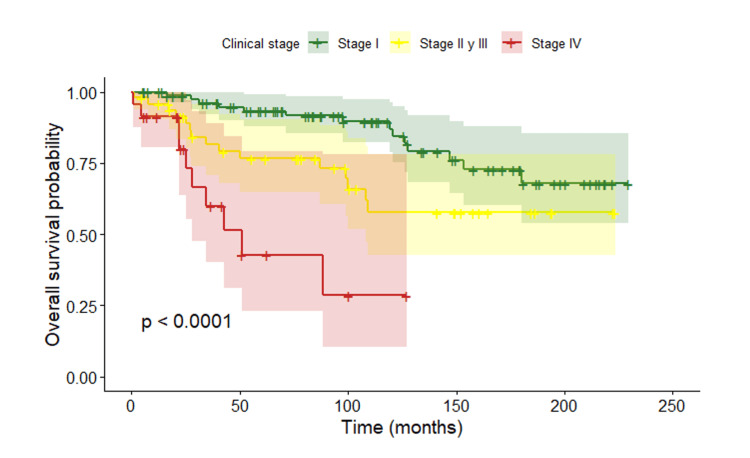
Overall survival according to clinical stage

Analysis of OS according to clinical stage showed statistically significant differences between groups (p < 0.0001) (Figure 2).

Patients diagnosed at stage I showed the most favorable survival, with few events during follow-up. A more pronounced decline in survival was observed in stages II-III after approximately 60 months, whereas patients with stage IV disease exhibited the poorest outcomes, with an early and marked decrease in survival.

The median OS was not reached, as more than 50% of patients remained alive at the end of follow-up. Estimated OS rates were approximately 87% at 5 years for stage I, 67% for stages II-III, and substantially lower for stage IV disease.

**Figure 3:**
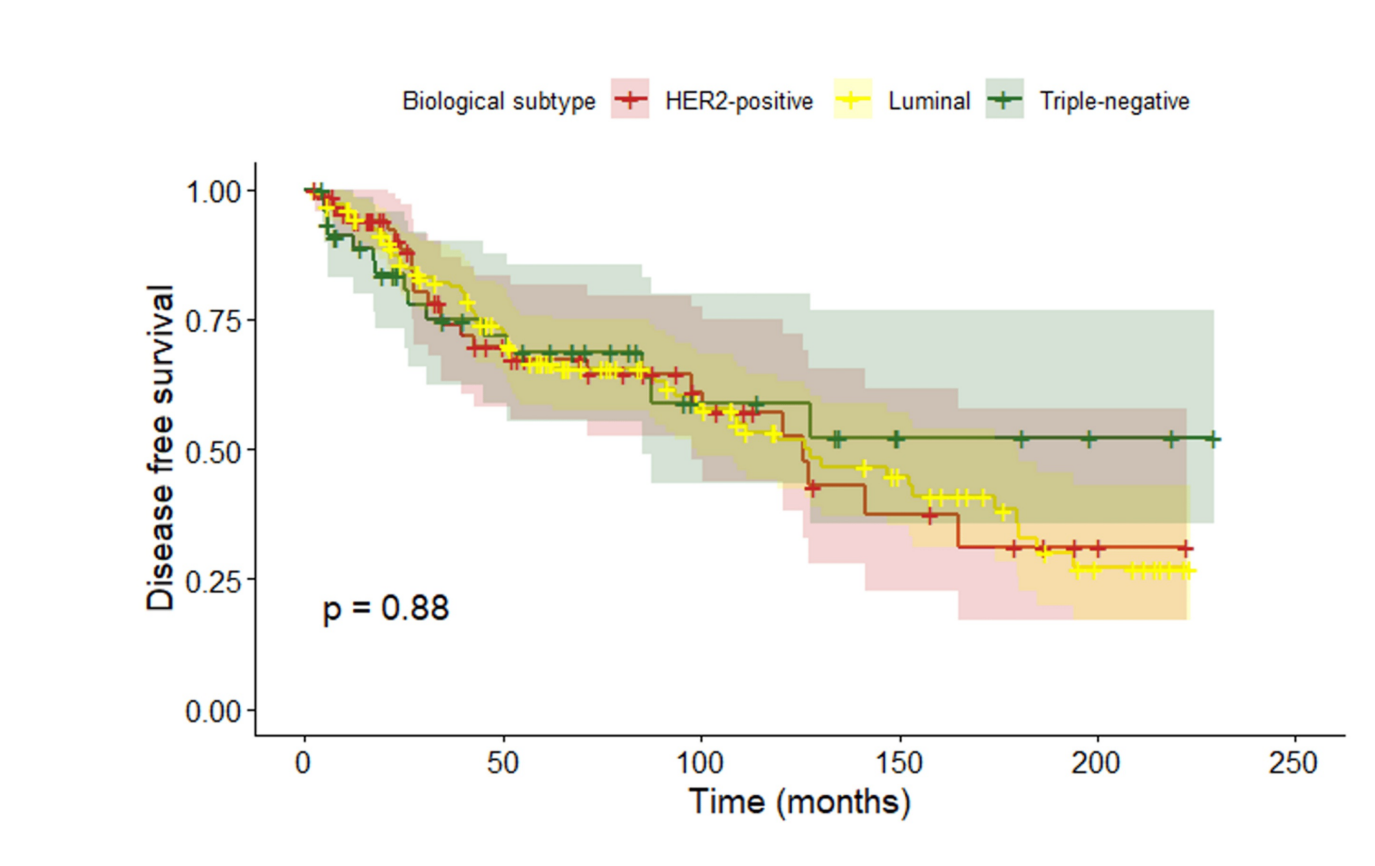
Disease-free survival according to biological subtype

The estimated DFS rate was approximately 73% at 5 years, decreasing to around 50% at 10 years. When DFS was analyzed according to biological subtype, no statistically significant differences were observed between groups (p = 0.877) (Figure 3).

**Figure 4:**
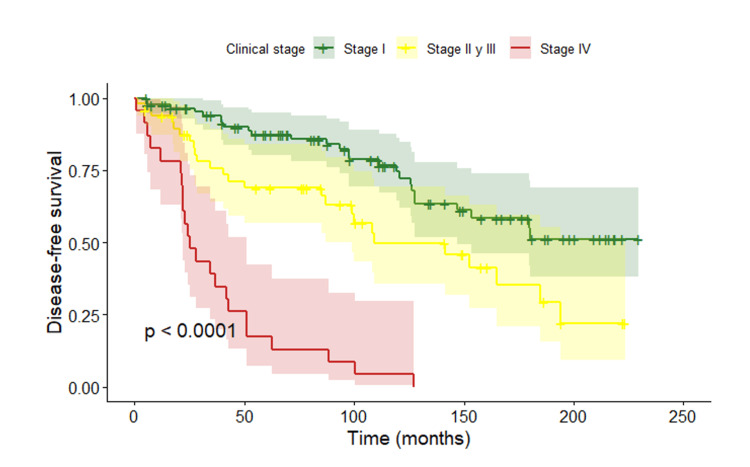
Disease-free survival according to clinical stage at diagnosis

In contrast, analysis of DFS according to clinical stage at diagnosis showed statistically significant differences between groups (p < 0.0001) (Figure 4).

## Discussion

The results of this study allow for the characterization of breast cancer in young women treated at a referral center and the analysis of their clinical, biological, and evolutionary features in the context of existing literature.

In this cohort of young women with breast cancer, a significant proportion of patients had a family history of cancer, consistent with reports from Latin American series and previous studies, reinforcing the role of hereditary factors in this subgroup (7%-31%) [14-17]. However, access to genetic testing was limited during the study period, highlighting a gap in the systematic identification of patients with hereditary cancer predisposition. This finding is particularly relevant for young women, given the importance of genetic evaluation for risk stratification, therapeutic decision-making, and follow-up planning.

Other known risk factors for breast cancer in young women, such as hormonal exposure (including oral contraceptive use), menstrual and reproductive history, obesity, lifestyle factors, and parity, were not systematically collected and therefore could not be analyzed in this study. These factors are known to interact with genetic susceptibility and may modulate cancer risk and disease expression. Importantly, the presence of germline pathogenic variants does not invariably result in clinical disease, highlighting the multifactorial nature of breast cancer development. Future prospective studies should integrate genetic, reproductive, hormonal, and lifestyle factors to better characterize risk profiles in young women.

Additionally, a higher frequency of second primary tumors was observed in our cohort compared with that reported in the literature, where the estimated 10-year risk is approximately 2.6% for contralateral breast cancer and 3% for non-breast malignancies [18-21]. In our cohort, second non-breast primary tumors predominated, a finding that may reflect greater, but not fully characterized, genetic susceptibility and the prolonged follow-up inherent to a young population. In young women, the development of second primary tumors has been associated with germline BRCA1/2 mutations, hereditary cancer predisposition syndromes, exposure to cytotoxic therapies or radiotherapy, and longer life expectancy after the initial diagnosis [18-21]. These findings underscore the importance of strengthening access to genetic counseling and integrating long-term surveillance strategies into the management of young women with breast cancer, particularly within public health systems and among socioeconomically vulnerable populations.

In low- and middle-income countries, including those in Latin America, a higher proportion of breast cancers are diagnosed at advanced stages, which is associated with poorer prognosis and increased mortality [22,23]. This trend appears to be even more pronounced in young women, as regional series report a high frequency of locally advanced or metastatic disease at diagnosis compared with older women [4,24]. In our cohort, stage II was the most frequent, followed by stage III, whereas stages I and IV accounted for a smaller proportion of cases (15.2%), indicating that breast cancer in young women tends to be diagnosed at more advanced stages than those reported in population-based registries (stage I 29%, stage IV 9%) [25]. The care profile of the included centers, belonging to the public health sector and serving a population with potentially greater socioeconomic vulnerability, may have influenced the stage at diagnosis.

In our cohort, invasive ductal carcinoma was the predominant histological type, with a predominance of high-grade tumors, consistent with international and regional literature that describes a higher proportion of grade III carcinomas in young women compared with older age groups [26-28]. This finding reinforces the notion of more aggressive tumor biology in this subgroup, with potential prognostic implications.

Regarding the distribution of biological subtypes, the luminal subtype was the most frequent, followed by HER2-positive and triple-negative disease, with a relatively higher proportion of aggressive subtypes among young women. This finding is consistent with both international [26-28] and national [25] reports and reflects a tumor profile associated with poorer prognosis in this age group [25-28].

Genomic platforms were used in a substantial proportion of patients (21%), including cases beyond classical indications (only 11% had luminal, HER2-negative tumors with negative axillary status). This highlights the need to optimize patient selection for these assays, particularly in settings with limited access such as Latin America, given their clinical impact and associated costs [29-31].

OS in our cohort fell within ranges reported internationally and regionally. With a median follow-up of 52.3 months, estimated OS was 84.8% at 5 years and 80.9% at 10 years, comparable to values observed in high-income countries [32,33]. In Latin America, 5-year OS shows greater heterogeneity, with rates ranging from 50.5% to 92.5%, depending on the country, treatment center, and tumor characteristics [26]. In regional multicenter studies, the 5-year OS for young women with stage II-III breast cancer has been reported to be approximately 83.9% [34].

Regarding biological subtypes, multiple studies have shown that young patients present a higher proportion of aggressive tumor subtypes, such as triple-negative and HER2-positive disease, compared with older women. However, the prognostic impact of young age depends on tumor subtype. In young women with luminal tumors (HR+/HER2−), age under 40 years has been independently associated with worse OS and DFS. In the literature, both OS and DFS vary according to biological subtype and stage at diagnosis [35,36]. In our cohort, no significant differences in OS or DFS were observed according to biological profile. This finding may be explained by the limited sample size, which reduces statistical power, and by the duration of follow-up, which may be insufficient to capture late recurrences in luminal tumors, whose risk persists beyond the first years after diagnosis. Additionally, the lack of precise discrimination between luminal A and luminal B subtypes may have contributed to an artificial homogenization of survival curves and attenuation of the expected differences between groups. In this study, disease recurrence was analyzed according to biological subtypes rather than histological subtypes, as biological classification is more closely related to prognosis and treatment decisions in breast cancer.

In contrast, stage at diagnosis showed a clear impact on both OS and DFS, with poorer outcomes observed at more advanced stages, consistent with widely reported literature [35,37]. Stage remains the main prognostic determinant in young women, regardless of biological subtype, particularly in locally advanced or metastatic disease [38].

Among the strengths of this study is the size of the cohort, which includes 267 young women with breast cancer, representing one of the largest series available at the national level for this age group. The availability of clinical, pathological, therapeutic, and survival outcome data allowed for a comprehensive analysis of prognostic factors in this population.

Among the limitations, the retrospective design should be acknowledged, as it relies on the quality and completeness of clinical records, resulting in missing data for some relevant variables, such as genetic and molecular studies. Surgical management was not based on a single standardized protocol throughout the study period; decisions regarding the type of surgery were made by multidisciplinary teams following national and international guidelines applicable at the time of diagnosis. Given the retrospective and multicenter nature of the study, surgeon-related variability in surgical decision-making could not be completely excluded. Additionally, the duration of follow-up may have limited the ability to detect survival differences among specific subgroups.

## Conclusions

This study characterized breast cancer in young women under 40 years of age in Uruguay, demonstrating a more aggressive clinical presentation, with a higher frequency of advanced-stage disease and biological subtypes associated with poorer prognosis compared with older women. Despite this, OS and DFS were comparable to those reported in international and regional series, suggesting appropriate therapeutic management in this cohort.

Stage at diagnosis emerged as the main prognostic determinant, while no significant differences in survival were observed according to biological subtype. These findings underscore the importance of early diagnosis, timely access to guideline-based treatments, and the strengthening of early detection strategies and multidisciplinary care to improve oncologic outcomes in young women with breast cancer.
